# DNA barcoding of commercially important reef fishes in Weh Island, Aceh, Indonesia

**DOI:** 10.7717/peerj.9641

**Published:** 2020-08-05

**Authors:** Nur Fadli, Siti Azizah Mohd Nor, Ahmad Sofiman Othman, Hizir Sofyan, Zainal A. Muchlisin

**Affiliations:** 1 Faculty of Marine and Fisheries, Syiah Kuala University, Banda Aceh, Aceh, Indonesia; 2 Institute of Marine Biotechnology, Universiti Malaysia Terengganu, Terengganu, Malaysia; 3 School of Biological Sciences, Universiti Sains Malaysia, Penang, Malaysia; 4 Faculty of Mathematics and Natural Science, Syiah Kuala University, Banda Aceh, Aceh, Indonesia

**Keywords:** DNA barcoding, Aceh, Reef fishes, Weh Island, Fisheries

## Abstract

Knowledge on the precise identification of fish resources is critical for sustainable fisheries management. This study employs the DNA barcoding approach to generate a molecular taxonomic catalogue of commercially important reef fishes in the waters of Weh Island (Aceh Province), the most northerly inhabited island in the biodiverse Indonesian Archipelago. The waters not only support artisanal fisheries but also a feeder for the industry in the greater island of Aceh. In total, 230 specimens from 72 species belonging to 32 genera and 17 families were DNA barcoded, representing a major segment of the captured reef fish taxa and a quarter of fish species diversity that had previously been recorded. The sequence read lengths were 639 bp revealing 359 conserved sites, 280 variable sites, 269 parsimony informative and 11 singletons. Our molecular findings paralleled the morphological identification with no evidence of cryptic species or new species discovery. This study is a significant contribution to the fisheries statistics of this area, which would facilitate assessment of species catch composition and hence for strategizing management plans. It is an important input to the DNA barcode library of Indonesian marine fishes and to the global DNA barcode entries in general.

## Introduction

Weh Island (5°50′N, 95°20′E) is located north of the main island of Sumatra, Indonesia and is within the province of Aceh (widely known for the 2004 Tsunami catastrophe). Surrounded by three great water bodies: the Andaman Sea, the Indian Ocean and the Straits of Malacca, Sumatra Island is rich in biodiversity including in its surrounding marine ecosystem (Brown, 2007). This amazing feature has attracted many scientific investigations. Baird et al. (2012) and Rudi et al. (2012a) reported that the Aceh waters around Weh Island might also be a hotspot for coral biodiversity comparable to the Coral Triangle. In particular, the mean species richness of the *Acropora* has been estimated to be comparable to regions within the Coral Triangle, an area centered in eastern Indonesia that is considered to be the most diverse marine region in the world (Rudi et al., 2012a). The high richness has been attributed to the area being a source of overlap of three distinct groups of faunas: widespread Indo-Pacific species, Indian Ocean species and Pacific Ocean species (Baird et al., 2012).

The coral ecosystem in Weh Island waters is home to no less than 350 reef fish species community (Campbell et al., 2005; Campbell et al., 2007; Rudi et al., 2008; Rudi et al., 2012b). Allen & Adrim (2003) documented six endemic reef fish species in this area. Indonesia is recognized to have the highest diversity of reef fishes species in the world with more than 2500 reef fish species recorded (Allen & Erdmann, 2012). This biodiversity is a very important source of livelihood to the Weh Island local artisanal fishermen. However, the bigger boats plying these waters transport their catch to other major landing sites in Aceh. The majority of fishes caught in this island are reef fishes, mainly groupers (Rudi et al., 2012b).

To ensure the perpetuity of this biodiversity, particularly with its relationship to the community well-being, it is imperative that management efforts are in place. A reliable species list is one of the most crucial baseline data in developing fisheries management plan and essential for conservation and sustainable exploitation (Basheer et al., 2017; Díaz et al., 2016; Pavan-Kumar et al., 2018; Ward, 2000). Earlier reports on reef fish identification in Weh Island had been based on morphological characters, and several species identification remains inconclusive (Rudi et al., 2012b). Precise species identification of landing catches is very important for capture fisheries management. In most major fisheries countries (including Indonesia), the majority of fish landing data are not recorded based on species names but typically the fishes are registered with a local name. For instance, in Aceh, Indonesia, the groupers are collectively recorded as ”kerapu” instead of the precise scientific species names. Another example is for some snappers in Malaysia. The Department of Fisheries Malaysia uses the term red snapper (”Merah”) to collectively refer to two species, *Lutjanus malabaricus* and *Lutjanus sebae*, while ”remong/kunyit-kunyit” is applied to species in the yellow-lined group including *Lutjanus lutjanus* and *Lutjanus vitta* (Bakar et al., 2018). It is impossible to develop effective management strategies and conservation plans without knowing what species are involved. Inaccurate species identification would undoubtedly affect the accuracy of stock inventories. This has severe implications on the ability to sustainably manage highly exploited species when pooled with species not under threat. Thus, a high proportion of misidentifications in the catch statistics could lead to considerable economic consequences.

Since its introduction in 2003, the DNA barcoding technique has become the golden standard for molecular taxonomy (Hebert et al., 2003). DNA barcoding has been recognized for its effectiveness in species identification for its rapid and robust output based on the cytochrome c oxidase subunit I (COI) gene. This technique has been utilized in species identification of marine fishes globally (Basheer et al., 2017; Bingpeng et al., 2018; Mat Jaafar et al., 2012; Ward et al., 2005; Weigt et al., 2012; Zhang & Hanner, 2011). It has a wide range of applications including identification of species that have similar morphological characters (Bingpeng et al., 2018), individuals at different developmental stages (Hubert et al., 2010), damaged and incomplete specimens (Wang et al., 2018), processed products i.e., food forensics (Chin Chin et al., 2016; Marko et al., 2004), cryptic species and new species discovery (Lim et al., 2016; Farhana et al., 2018).

There have been a few documentations of molecular studies of freshwater fishes of Aceh e.g., DNA barcode of freshwater fishes in Lake Laut Tawar Aceh (Muchlisin, Fadli & Siti-Azizah, 2012), the genetic diversity and structure of the striped snakehead (Tan et al., 2015) DNA barcode of freshwater halfbeak genus *Hemirhamphodon* from Sundaland (Lim et al., 2016), DNA barcode of tropical eels of Aceh (Muchlisin et al., 2017), and DNA barcode of freshwater halfbeak genus *Dermogenys* in Southeast Asia (Farhana et al., 2018). However, there is no record of reef fish species identification through DNA barcoding in Aceh. Related studies in the Indonesian waters have been coincidental as part of larger projects over a wider coverage, with limited representative specimens from the Aceh region (Anzani et al., 2019; Madduppa et al., 2017; Madduppa et al., 2016; Prehadi et al., 2015; Sembiring et al., 2015; Wardiatno et al., 2015; Wibowo et al., 2018). Thus, this investigation is the first comprehensive study of DNA barcoding of commercially important reef fishes in Indonesia, with focus on the marine waters of Weh Island. It would also be an important contribution to the global DNA barcode library and will facilitate fisheries management and conservation programmes in Weh Island specifically, and the Aceh waters (and Indonesia) in general.

## Materials & Methods

### Sample collection

**Figure 1:**
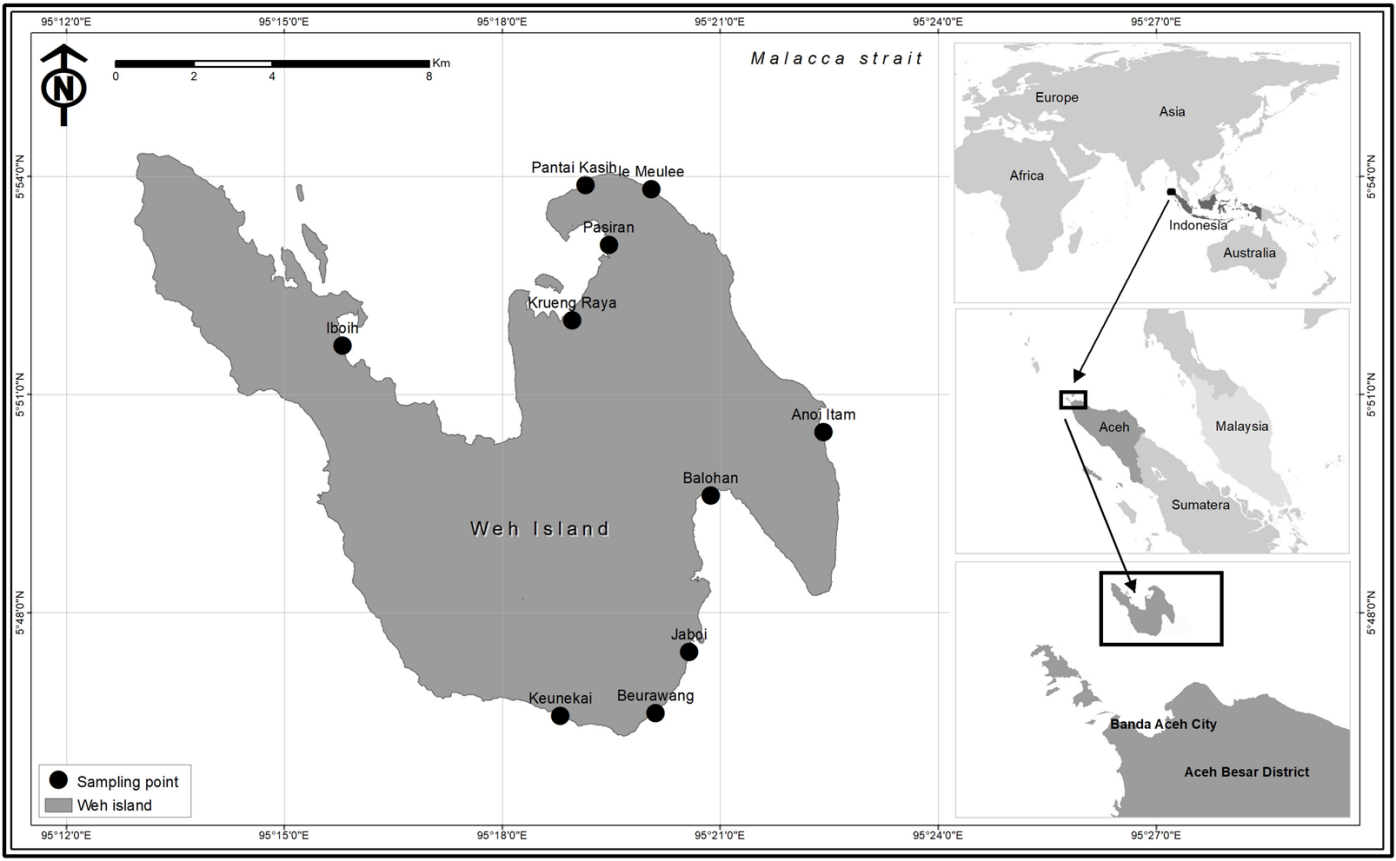
Map of Weh Island, Aceh, Indonesia showing the sampling sites.

A total of 230 coral reef fish specimens were collected from fish landing sites and fish markets located throughout Weh Island (except for the west coast of Weh Island since this is a protected forest area, with no fish landing sites permitted) from May 2015 to November 2016 (Fig. 1). Reef fishes were selected from the total landings based on the following taxonomic keys: Heemstra & Randall (1993), Craig, Mitcheson & Heemstra (2011), and Froese & Pauly (2019). Each sample was documented according to the requirements of the BOLD Systems, including digital specimen photograph. Fin biopsy was performed at the collection sites. Tissue samples were fixed with 96% ethanol placed in individual two mL microcentrifuge tube. Sample sizes of each morphospecies ranged from 1 to 9 individuals with an average of 3 specimens classified into 72 presumed species (53 species were represented by more than one specimen while 19 species by only a single individual) depending on the availability of the species. Field experiments were approved by the Faculty of Marine and Fisheries, Syiah Kuala University, Banda Aceh, Indonesia (16030/A2.1/KP/2016). The committee of Research Ethics and Animal Care of Syiah Kuala University provided full approval for this research (Ethic Code No: 958/2015). Voucher specimens have been deposited at the Faculty of Marine and Fisheries, Syiah Kuala University at Banda Aceh, Indonesia. No specific authorization were required for sampling since samples were obtained from commercial fishers and fish markets. In many cases, tissues were contributed by the fishers. For those samples which lack morphological vouchers (big fishes), “photographic voucher” were obtained following the Fish-BOL collaborator’s protocol (Steinke & Hanner, 2011). All DNA sequences have been deposited in BOLD systems under the project “DNA barcoding of commercially important reef fishes in Weh Island, Aceh” (http://www.boldsystems.org/). Other supplementary data of the collected fish specimens, including their BOLD Accession numbers, are presented in Table 1.

**Table 1:** List of the studied fish species from Weh Island, Aceh, Indonesia, and their BOLD accession numbers.

**BOLD Accession No.**	**Family**	**Species**	**n**
FADLI001-17–FADLI005-17	Acanthuridae	*Acanthurus mata*	5
FADLI010-17		*Acanthurus triostegus*	1
FADLI011-17–FADLI014-17		*Acanthurus xanthopterus*	4
FADLI015-17–FADLI017-17	Apogonidae	*Apogon novemfasciatus*	3
FADLI141-17–FADLI142-17	Balistidae	*Melichthys indicus*	2
FADLI167-17–FADLI170-17		*Odonus niger*	4
FADLI187-17		*Rhinecanthus verrucosus*	1
FADLI227-17–FADLI228-17		*Sufflamen fraenatum*	2
FADLI019-17–FADLI021-17	Caesionidae	*Caesio xanthonota*	3
FADLI022-17	Epinephelidae	*Cephalopholis argus*	1
FADLI023-17–FADLI027-17		*Cephalopholis aurantia*	5
FADLI028-17–FADLI032-17		*Cephalopholis formosa*	5
FADLI033-17		*Cephalopholis leopardus*	1
FADLI034-17–FADLI038-17		*Cephalopholis miniata*	5
FADLI039-17–FADLI040-17, FADLI048-17–FADLI056-17		*Cephalopholis nigripinnis*	11
FADLI041-17–FADLI047-17		*Cephalopholis sonnerati*	7
FADLI062-17–FADLI063-17		*Epinephelus areolatus*	2
FADLI064-17–FADLI065-17		*Epinephelus coeruleopunctatus*	2
FADLI066-17–FADLI072-17		*Epinephelus fasciatus*	7
FADLI073-17–FADLI074-17		*Epinephelus longispinis*	2
FADLI075-17–FADLI078-17		*Epinephelus melanostigma*	4
FADLI079-17–FADLI084-17		*Epinephelus merra*	6
FADLI085-17		*Epinephelus spilotoceps*	1
FADLI086-17–FADLI087-17		*Epinephelus tauvina*	2
FADLI088-17		*Epinephelus undulosus*	1
FADLI230-17–FADLI233-17		*Variola albimarginata*	4
FADLI234-17–FADLI235-17		*Variola louti*	2
FADLI182-17–FADLI184-17	Haemulidae	*Plectorhinchus vittatus*	3
FADLI185-17		*Plectorhinchus chaetodonoides*	1
FADLI147-17–FADLI151-17	Holocentridae	*Myripristis adusta*	5
FADLI152-17–FADLI159-17		*Myripristis berndti*	7
FADLI161-17		*Myripristis murdjan*	1
FADLI162-17–FADLI165-17		*Myripristis violacea*	4
FADLI166-17		*Neoniphon argenteus*	1
FADLI188-17–FADLI191-17		*Sargocentron caudimaculatum*	4
FADLI192-17–FADLI195-17		*Sargocentron diadema*	4
FADLI196-17		*Sargocentron punctatissimum*	1
FADLI102-17–FADLI103-17	Kyphosidae	*Kyphosus cinerascens*	2
FADLI018-17	Labridae	*Bodianus mesothorax*	1
FADLI057-17–FADLI060-17		*Cheilinus trilobatus*	4
FADLI092-17–FADLI093-17		*Halichoeres hortulanus*	2
FADLI094-17		*Hemigymnus melapterus*	1
FADLI229-17		*Thalassoma jansenii*	1
FADLI089-17–FADLI091-17	Lethrinidae	*Gnathodenthex aureolineatus*	3
FADLI109-17–FADLI111-17		*Lethrinus conchyliatus*	3
FADLI105-17–FADLI107-17		*Lethrinus ornatus*	3
FADLI112-17–FADLI116-17		*Lethrinus rubrioperculatus*	5
FADLI117-17–FADLI119-17	Lutjanidae	*Lutjanus decussatus*	3
FADLI120-17–FADLI121-17		*Lutjanus jhonii*	2
FADLI122-17–FADLI126-17		*Lutjanusbengalensis*	5
FADLI127-17–FADLI132-17		*Lutjanus lutjanus*	6
FADLI133-17–FADLI140-17		*Lutjanus rufolineatus*	8
FADLI143-17–FADLI146-17	Mullidae	*Mulloidichthys vanicolensis*	4
FADLI171-17–FADLI174-17		*Parupeneus barberinus*	2
FADLI175-17–FADLI177-17		*Parupeneus macronemus*	3
FADLI219-17–FADLI223-17	Nemipteridae	*Scolopsis affinis*	5
FADLI224-17–FADLI226-17		*Scolopsis bilineata*	3
FADLI178-17–FADLI179-17	Pempheridae	*Pempheris flavicycla*	2
FADLI180-17		*Pempheris vanicolensis*	1
FADLI006-17	Pomacentridae	*Abudefduf septemfasciatus*	1
FADLI007-17–FADLI009-17		*Abudefduf vaigiensis*	3
FADLI095-17–FADLI101-17	Priacanthidae	*Heteropriacanthus carolinus*	7
FADLI186-17		*Priacanthus hamrur*	1
FADLI061-17	Scaridae	*Chlorurus sordidus*	1
FADLI236-17–FADLI237-17		*Chlorurus strongylocephalus*	2
FADLI197-17		*Scarus frenatus*	1
FADLI198-17–FADLI199-17		*Scarus ghobban*	2
FADLI200-17–FADLI208-17		*Scarus niger*	9
FADLI212-17–FADLI214-17		*Scarus prasiognathos*	3
FADLI215-17–FADLI217-17		*Scarus quoyi*	3
FADLI209-17–FADLI211-17		*Scarus rubroviolaceus*	3
FADLI218-17		*Scarus spinus*	1
**Total**	**17**	**72**	**230**

**Notes.**

n, number of sequences.

### DNA extraction, primer, and PCR assay

The modified Cetyltrimethyl Ammonium Bromide (CTAB) protocol was used to isolate genomic DNA (Bakar et al., 2018). The extracted DNA was quantified using the Nano Drop 2000c Spectrophotometer (Thermo Scientific, Waltham, MA). The COI gene was amplified using the universal primer sets of Ward et al. (2005). PCR was set up in a 25 µL mixture reaction and was amplified in a BIO-RAD T100 Thermal Cycler (BioRad Laboratories Inc., USA). The total reaction volume of 25 µL contained 2.0 µL DNA template, 0.5 µL of each primer, 2.5 µL of 10x *i*-Taq™ plus PCR Buffer, 2.0 of 25 mM MgCl_2_, 1.0 µL of dNTP, 0.25µL of *i*-Taq™ plus DNA Polymerase and 16.25 µL of Milli-Q water. The thermal conditions consisted of an initial denaturation at 95 °C for 2 min followed by 30 cycles of denaturation at 94 °C for 45 s; annealing at 47.9–60 °C for 45 s; elongation at 72 °C for 1 min and a final extension of 72 °C for 10 min before termination of the reaction at 4 °C.

### Gel electrophoresis, staining, and DNA sequencing

Amplicons were separated and visualized on a 1.7% agarose gel stained with 2 to 2.5 µL of RedSafe™ Nucleic Acid Staining Solution (IntRON Biotechnology, Gyeonggi-do, Korea). A 2.0 µL volume of PCR product was loaded onto the agarose gel and electrophoresed at 100V for 30 min to assess its quality. Satisfactory PCR products were sent to the First BASE Laboratories, Malaysia, for bidirectional sequencing using BigDye Terminator v3.1 Cycle Sequencing Kit and ABI PRISM 3730xl DNA Analyzer (Applied Biosystems, Foster City, CA) following manufacturer’s protocol.

### Data analysis

Forward and reverse COI sequences were aligned in Clustal W and edited in MEGA 6.06 (Tamura et al., 2013). To ensure accurate alignment and detection of stop codons if present, the aligned sequences were translated into proteins. To confirm their genetic identity, the aligned sequences were compared with previously known sequences and closely related species in BLAST (Basic Local Alignment Search Tool; http://www.ncbi.nlm.nih.gov/BLAST) and the BOLD Identification System (IDS) (http://www.boldsystems.org/).

Pairwise genetic distances at different taxonomic levels (within species, within genus, within family) were estimated based on the Kimura-2-parameter (K2P) model using MEGA 6.06 (Kimura, 1980; Tamura et al., 2013) and Sequence Analysis Engine of BOLD (http://www.boldsystems.org/). The MEGA 6.06 software was also used to cluster COI haplotypes into a Neighbour-Joining (NJ) phylogeny, employing 1000 bootstrap replicates. Furthermore, DnaSP 5.10 was used to summarize haplotype distributions (Librado & Rozas, 2009; Rozas et al., 2003).

Comparisons at the species level of the maximum intraspecific genetic distance with the minimum distance to the nearest neighbour were performed by applying the BOLD’s ‘Barcoding Gap Analysis’ tool (http://www.boldsystems.org). The Automatic Barcode Gap Discovery (ABGD) (with default settings and the K2P model) was used for species delimitation to determine the number of operational taxonomic units (OTUs) based on pairwise sequence distances between individuals within the dataset (https://bioinfo.mnhn.fr/abi/public/abgd/abgdweb.html) (Puillandre et al., 2012).

Further to the molecular work, the conservation status of the identified reef fishes was determined based on the International Union for Conservation of Nature (IUCN) website (http://www.iucnredlist.org, accessed on 13 April 2020). The IUCN criteria are widely accepted for assessing species conservation status (Hoffmann et al., 2008) including in the evaluation of the conservation status of plants (Callmander, Schatz & Lowry, 2005), corals (Carpenter et al., 2008), sea cucumbers (Madduppa et al., 2017) and several fish species (Morris, Roberts & Hawkins, 2000; Sadovy de Mitcheson et al., 2013), among others. The IUCN categorizes the conservation status into four groups namely: Threatened (T), Near Threatened (NT), Data Deficient (DD) and Least Concern (LC) (further description of each category can be found in http://www.iucnredlist.org). Besides their conservation status, their trade status was also determined using CITES (Convention on International Trade in Endangered Species) online at http://www.cites.org/ (accessed on 13 April 2020).

## Results

### Cytochrome oxidase subunit I diversity assessment

**Figure 2:**
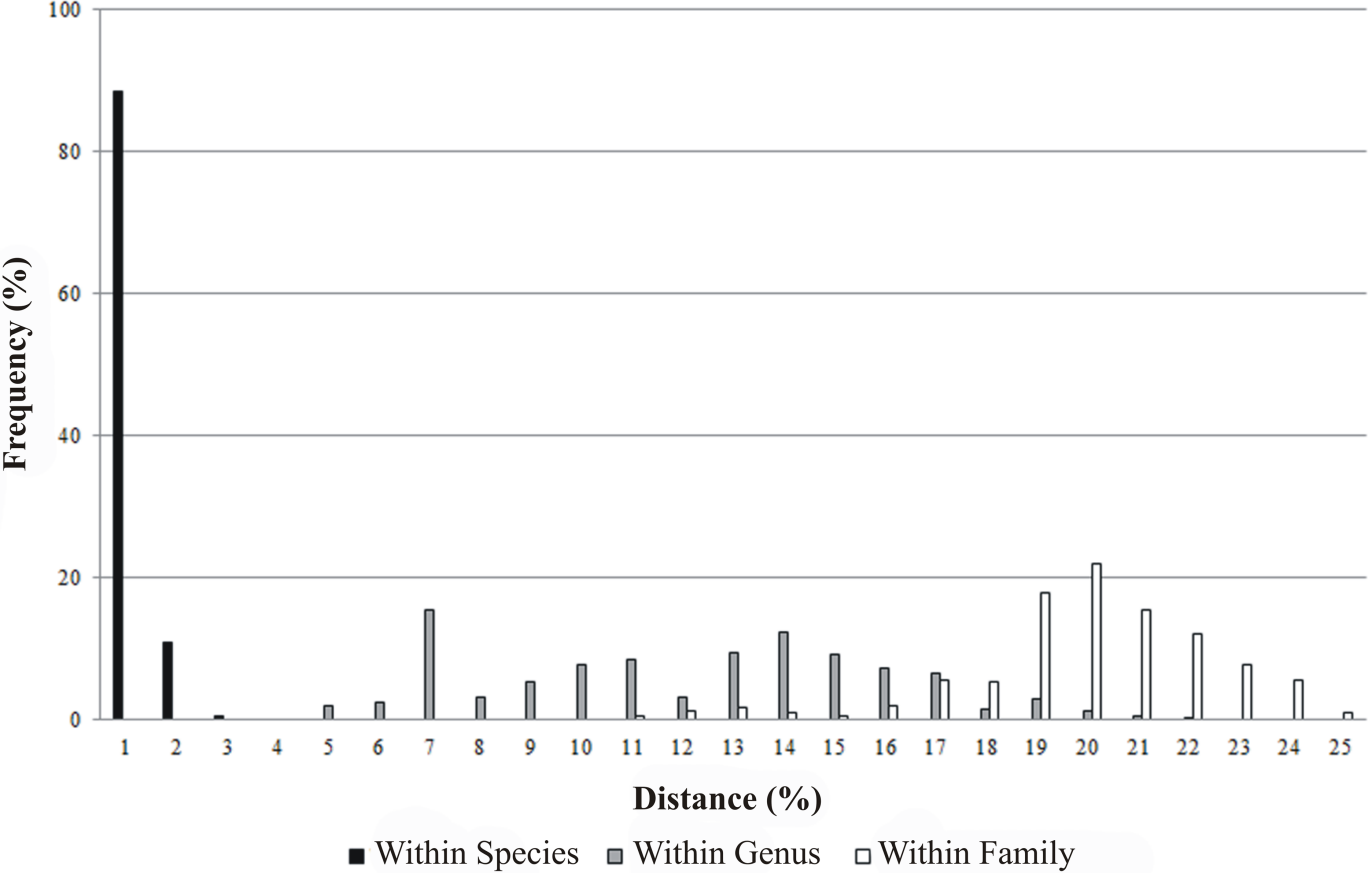
Frequency distribution of genetic distances (%) for COI within species, genus and family.

All 230 specimens were successfully sequenced generating 146 haplotypes. The sequence read lengths were 639 bp with average nucleotide composition of *A* = 23.78%, *T* = 29.58%, *C* = 28.39%, *G* = 18.25%. There were 359 conserved sites and 280 variable sites, of which 269 were parsimony informative and 11 singletons. The base composition showed that the AT content (53.36%) was higher than the GC content (46.64%). The mean T content was the highest, and the mean G content was the lowest. The GC content decreased in the order of first, second, to the third position codon with mean values of 57.09%, 42.70% and 40.14% respectively. Of the 230 COI sequences, no insertions, deletions or stop codons were detected in the samples. In addition, intra-taxon K2P genetic distances increased from lower to higher taxonomic levels: 0% to 1.55% within species, 3.79% to 20.73% between species within genus and 9.79% to 23.94% between genera within family. The mean K2P genetic distance between specimens was 0.24% within species, 11.16% between species within genus and 19.01% between genera within family (Fig. 2, Table 2). The average K2P genetic distance between species within genus was 46.5 fold higher than the average K2P genetic distance within species.

**Table 2:** Summary of genetic divergences (K2P percent) within and between various taxonomic levels based on the studied fish species from Weh Island, Aceh, Indonesia.

	*n*	Taxa	Comparisons	Minimum (%)	Mean (%)	Maximum (%)
Within Species	211	53	420	0	0.24	1.55
Within Genus	188	15	1428	3.79	11.16	20.73
Within Family	169	8	1679	9.76	19.01	23.94

### Species delimitation

**Figure 3:**
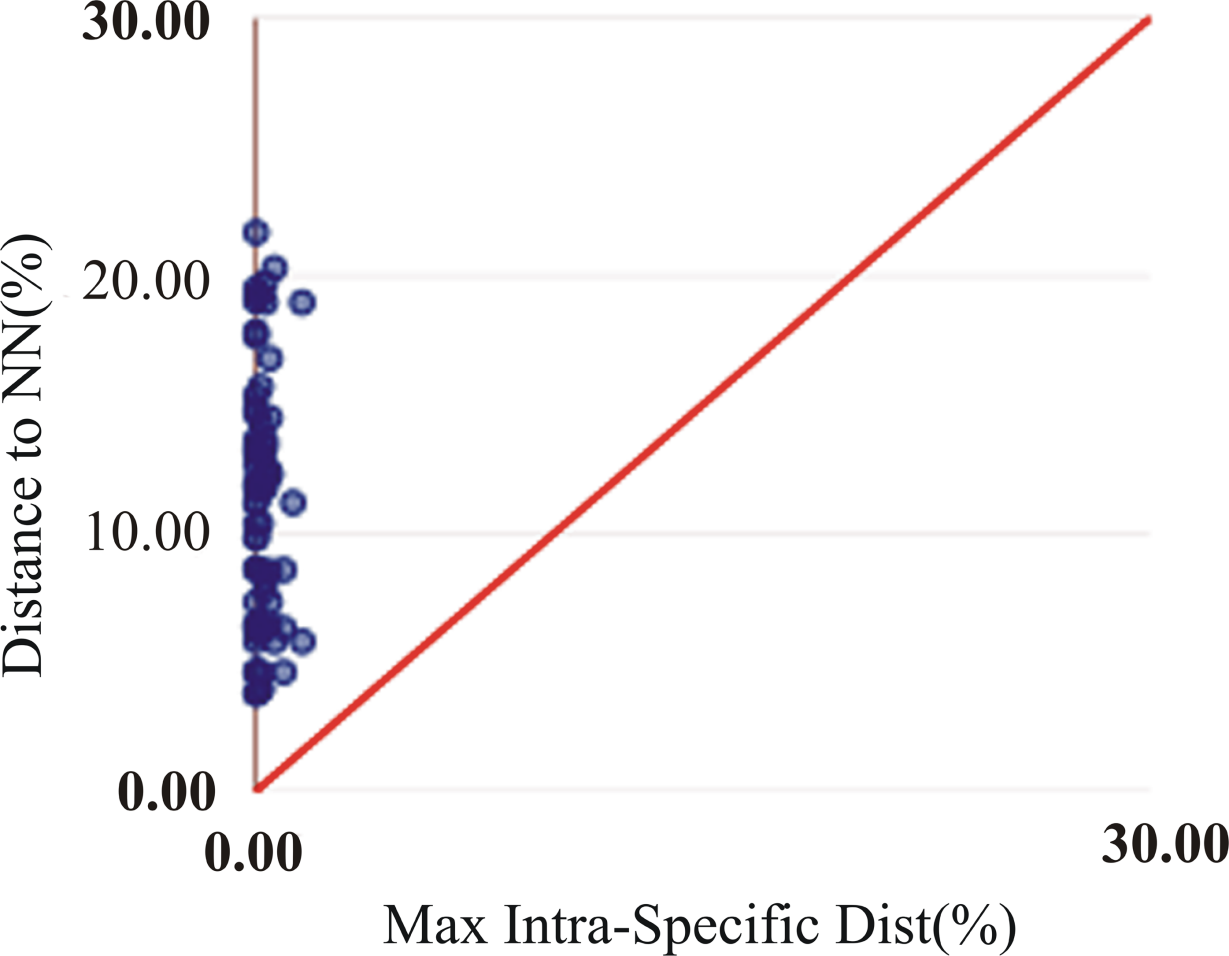
Maximum intraspecific divergence (% K2P) in the barcode region of COI plotted against nearest neighbour distance (% K2P) for the 72 species examined in this study. All comparisons had a barcode gap based on positions of all points above the red line. All comparisons had a barcode gap based on positions of all points above the red line.

**Figure 4:**
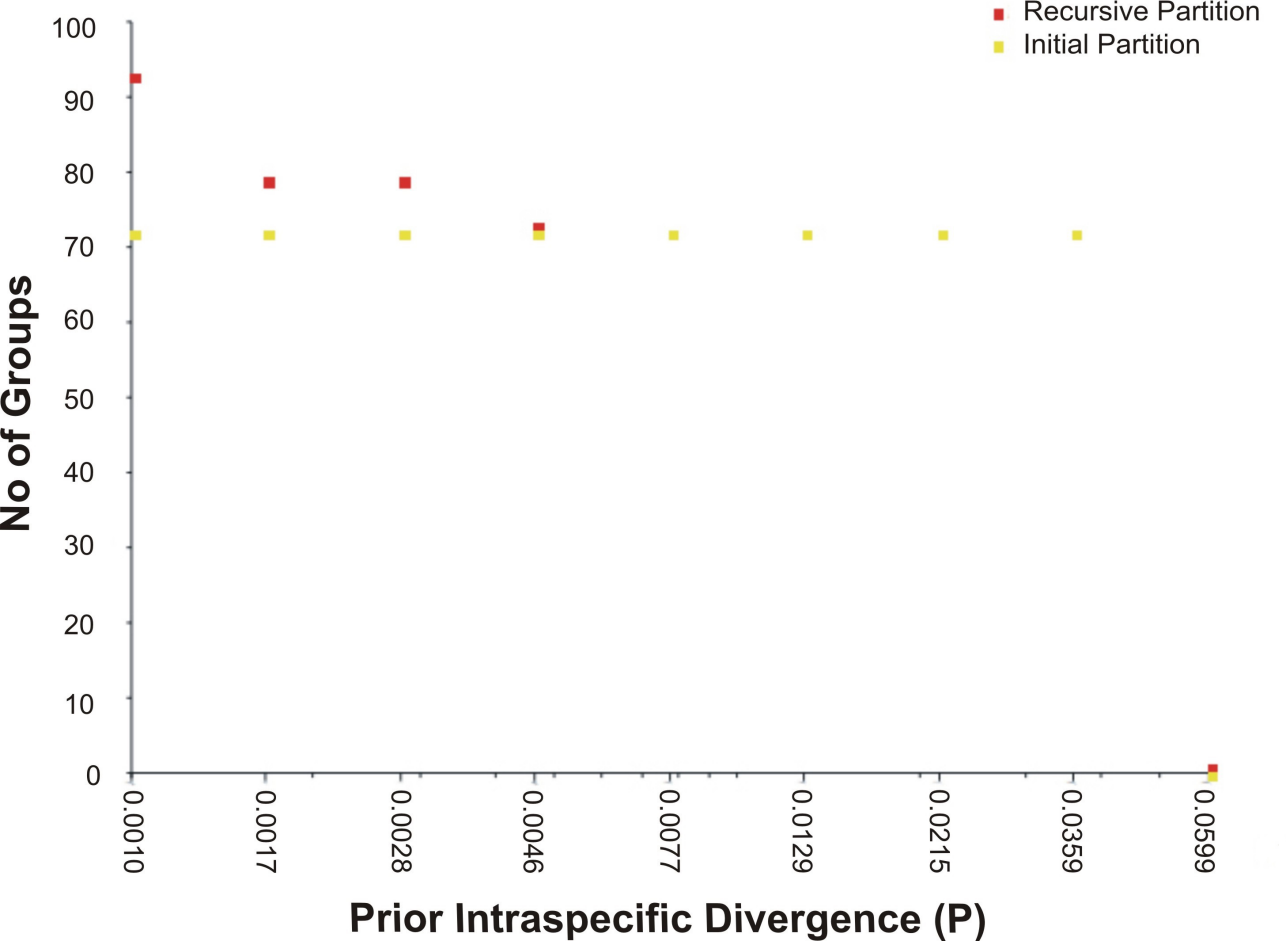
The number of genetically distinct OTUs according to the prior intraspecific divergence value generated by ABGD based on K2P.

**Figure 5:**
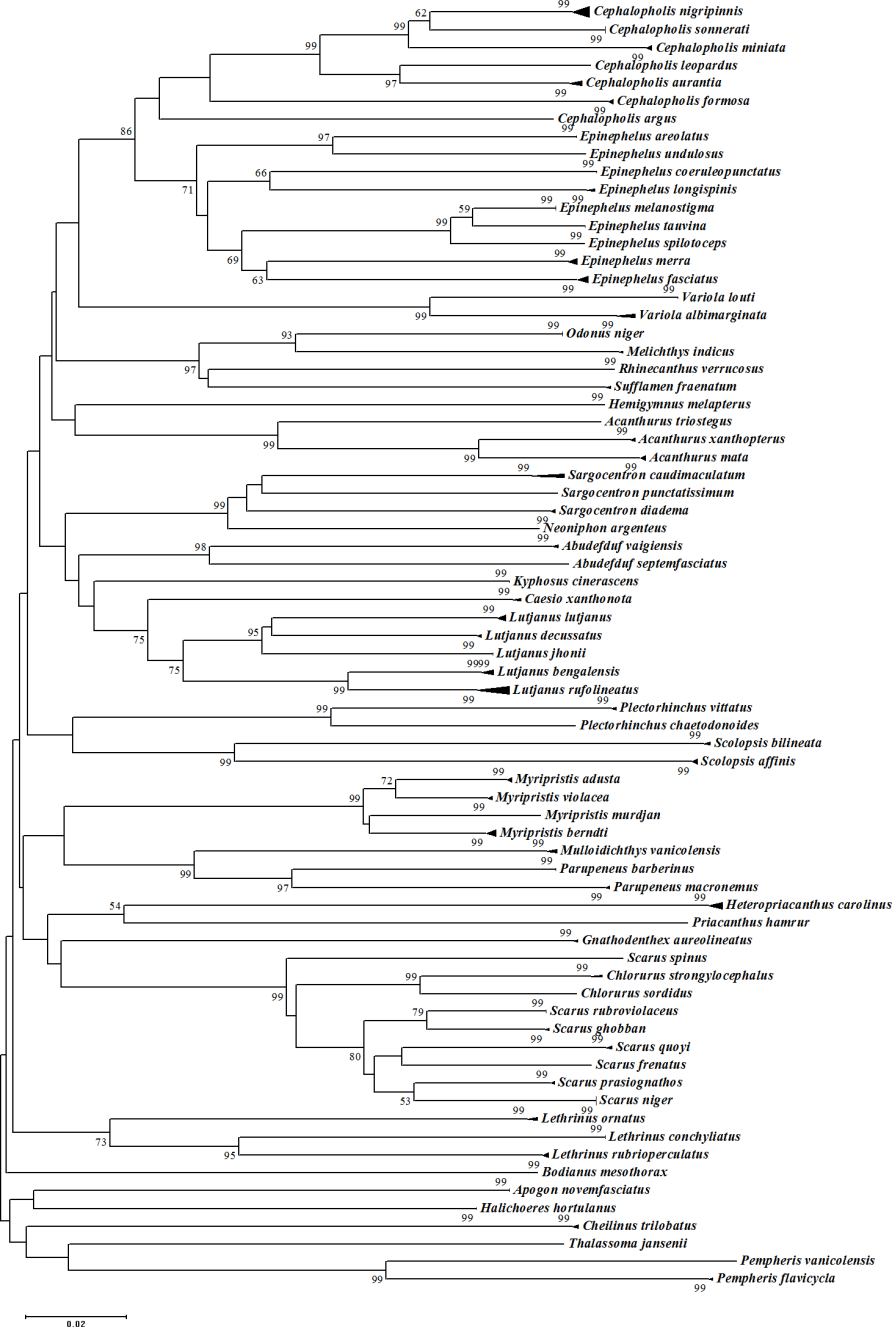
Neighbour-Joining (NJ) tree of COI barcodes for all samples. Bootstrap values <50% are not shown and scale bar indicates percent divergence calculated under the K2P model. Solid triangles represent clusters of multiple specimens (height is proportional to specimen number and the horizontal width is proportional to the genetic variation within each cluster).

The assessment of species identities with previously known sequences and closely related species in BLAST and BOLD databases yielded 98–100% identities indicating the efficacy of COI sequences to provide species-level resolution. In addition, Barcoding Gap Analysis showed that all putative species had a maximum intraspecies distance of less than 2%. The mean distance to the nearest neighbour (NN) was 11.13%. The latter analysis also showed that all specimens exhibited high distance values to their nearest neighbour indicating the presence of “barcode gap” among the 72 observed species (Fig. 3, Table 3). However, it should also be noted that there were several single-specimen species (not applicable for this analysis) and numerous NN that were not congenerics. In concordance with Barcoding Gap Analysis, ABGD also generated 72 operational taxonomic units (OTUs) with the initial partition at a prior intraspecific divergence (*P*) (*P* = 0.0077–0.03594) (Fig. 4). Furthermore, the NJ tree showed that all the recognized species formed monophyletic clusters without any overlap between species. All assemblages of conspecific individuals had a bootstrap support of 99% and 1.00 posterior probability (Fig. 5). Congeneric samples were also well resolved.

**Table 3:** The mean and maximum intraspecific values for each species, compared to the nearest neighbour distance. Where the species is a singleton, N/A is displayed for intraspecific values.

**No**	**Species**	**Mean intra-species (K2P%)**	**Max intra-species (K2P%)**	**Nearest neighbour**	**Distance to nearest neighbour (K2P%)**
1	*Acanthurus mata*	0.12	0.31	*Acanthurus xanthopterus*	6.3
2	*Acanthurus triostegus*	N/A	0	*Acanthurus xanthopterus*	13.23
3	*Acanthurus xanthopterus*	0.08	0.15	*Acanthurus mata*	6.3
4	*Myripristis adusta*	0.09	0.15	*Myripristis violacea*	3.95
5	*Myripristis berndti*	0.38	0.92	*Myripristis violacea*	4.61
6	*Myripristis murdjan*	N/A	0	*Myripristis berndti*	5.8
7	*Myripristis violacea*	0.08	0.15	*Myripristis adusta*	3.95
8	*Neoniphon argenteus*	N/A	0	*Sargocentron punctatissimum*	11.98
9	*Sargocentron caudimaculatum*	0.67	1.23	*Sargocentron punctatissimum*	11.19
10	*Sargocentron diadema*	0.08	0.15	*Sargocentron punctatissimum*	11.71
11	*Sargocentron punctatissimum*	N/A	0	*Sargocentron caudimaculatum*	11.19
12	*Apogon novemfasciatus*	0	0	*Lutjanus bengalensis*	17.64
13	*Bodianus mesothorax*	N/A	0	*Halichoeres hortulanus*	19.02
14	*Cheilinus trilobatus*	0.15	0.31	*Lutjanus bengalensis*	19.74
15	*Halichoeres hortulanus*	0	0	*Apogon novemfasciatus*	17.84
16	*Hemigymnus melapterus*	N/A	0	*Halichoeres hortulanus*	19.5
17	*Thalassoma jansenii*	N/A	0	*Epinephelus melanostigma*	19.24
18	*Chlorurus sordidus*	N/A	0	*Chlorurus strongylocephalus*	6.46
19	*Chlorurus strongylocephalus*	0.47	0.47	*Chlorurus sordidus*	6.46
20	*Scarus frenatus*	N/A	0	*Scarus prasiognathos*	6.47
21	*Scarus ghobban*	0.15	0.15	*Scarus rubroviolaceus*	4.62
22	*Scarus niger*	0	0	*Scarus prasiognathos*	6.17
23	*Scarus prasiognathos*	0.1	0.15	*Scarus niger*	6.17
24	*Scarus quoyi*	0.2	0.31	*Scarus frenatus*	7.83
25	*Scarus rubroviolaceus*	0	0	*Scarus ghobban*	4.62
26	*Scarus spinus*	N/A	0	*Scarus quoyi*	11.18
27	*Abudefduf septemfasciatus*	N/A	0	*Abudefduf vaigiensis*	13.62
28	*Abudefduf vaigiensis*	0.2	0.31	*Abudefduf septemfasciatus*	13.62
29	*Caesio xanthonota*	0.2	0.31	*Lutjanus lutjanus*	12.5
30	*Plectorhinchus chaetodonoides*	N/A	0	*Plectorhinchus vittatus*	10.38
31	*Plectorhinchus vittatus*	0.1	0.15	*Plectorhinchus chaetodonoides*	10.38
32	*Kyphosus cinerascens*	0	0	*Lutjanus bengalensis*	14.87
33	*Lutjanus decussatus*	0.1	0.15	*Lutjanus lutjanus*	8.53
34	*Lutjanus johnii*	0	0	*Lutjanus decussatus*	8.67
35	*Lutjanus bengalensis*	0.25	0.62	*Lutjanus rufolineatus*	5.8
36	*Lutjanus lutjanus*	0.36	0.46	*Lutjanus decussatus*	8.53
37	*Lutjanus rufolineatus*	0.66	1.55	*Lutjanus bengalensis*	5.8
38	*Mulloidichthys vanicolensis*	0.23	0.46	*Parupeneus barberinus*	14.49
39	*Parupeneus barberinus*	0	0	*Parupeneus macronemus*	11.78
40	*Parupeneus macronemus*	0.2	0.31	*Parupeneus barberinus*	11.78
41	*Pempheris flavicycla*	0.15	0.15	*Pempheris vanicolensis*	13.07
42	*Pempheris vanicolensis*	N/A	0	*Pempheris flavicycla*	13.07
43	*Heteropriacanthus carolinus*	0.41	0.62	*Neoniphon argenteus*	20.31
44	*Priacanthus hamrur*	N/A	0	*Lutjanus rufolineatus*	21.69
45	*Cephalopholis argus*	N/A	0	*Cephalopholis formosa*	15.35
46	*Cephalopholis aurantia*	0.18	0.46	*Cephalopholis leopardus*	7.33
47	*Cephalopholis formosa*	0.06	0.15	*Cephalopholis aurantia*	14.31
48	*Cephalopholis leopardus*	N/A	0	*Cephalopholis aurantia*	7.33
49	*Cephalopholis miniata*	0.13	0.31	*Cephalopholis sonnerati*	8.27
50	*Cephalopholis nigripinnis*	0.36	0.93	*Cephalopholis sonnerati*	6.31
51	*Cephalopholis sonnerati*	0	0	*Cephalopholis nigripinnis*	6.31
52	*Epinephelus areolatus*	0	0	*Epinephelus undulosus*	9.83
53	*Epinephelus coeruleopunctatus*	0	0	*Epinephelus longispinis*	12.67
54	*Epinephelus fasciatus*	0.2	0.46	*Epinephelus merra*	12.37
55	*Epinephelus longispinis*	0.31	0.31	*Epinephelus areolatus*	12.64
56	*Epinephelus melanostigma*	0	0	*Epinephelus tauvina*	3.79
57	*Epinephelus merra*	0.31	0.46	*Epinephelus tauvina*	12.24
58	*Epinephelus spilotoceps*	N/A	0	*Epinephelus tauvina*	4.61
59	*Epinephelus tauvina*	0	0	*Epinephelus melanostigma*	3.79
60	*Epinephelus undulosus*	N/A	0	*Epinephelus areolatus*	9.83
61	*Variola albimarginata*	0.67	0.93	*Variola louti*	8.58
62	*Variola louti*	0	0	*Variola albimarginata*	8.58
63	*Gnathodentex aureolineatus*	0.1	0.15	*Halichoeres hortulanus*	19.27
64	*Lethrinus conchyliatus*	0	0	*Lethrinus rubrioperculatus*	13.35
65	*Lethrinus ornatus*	0.31	0.46	*Lethrinus conchyliatus*	16.79
66	*Lethrinus rubrioperculatus*	0.12	0.31	*Lethrinus conchyliatus*	13.35
67	*Scolopsis affinis*	0.12	0.31	*Scolopsis bilineata*	18.97
68	*Scolopsis bilineata*	1.03	1.54	*Scolopsis affinis*	18.97
69	*Melichthys indicus*	0.15	0.15	*Odonus niger*	11.81
70	*Odonus niger*	0	0	*Melichthys indicus*	11.81
71	*Rhinecanthus verrucosus*	N/A	0	*Odonus niger*	14.66
72	*Sufflamen fraenatum*	0.15	0.15	*Odonus niger*	15.7

### Composition of fishes

**Figure 6:**
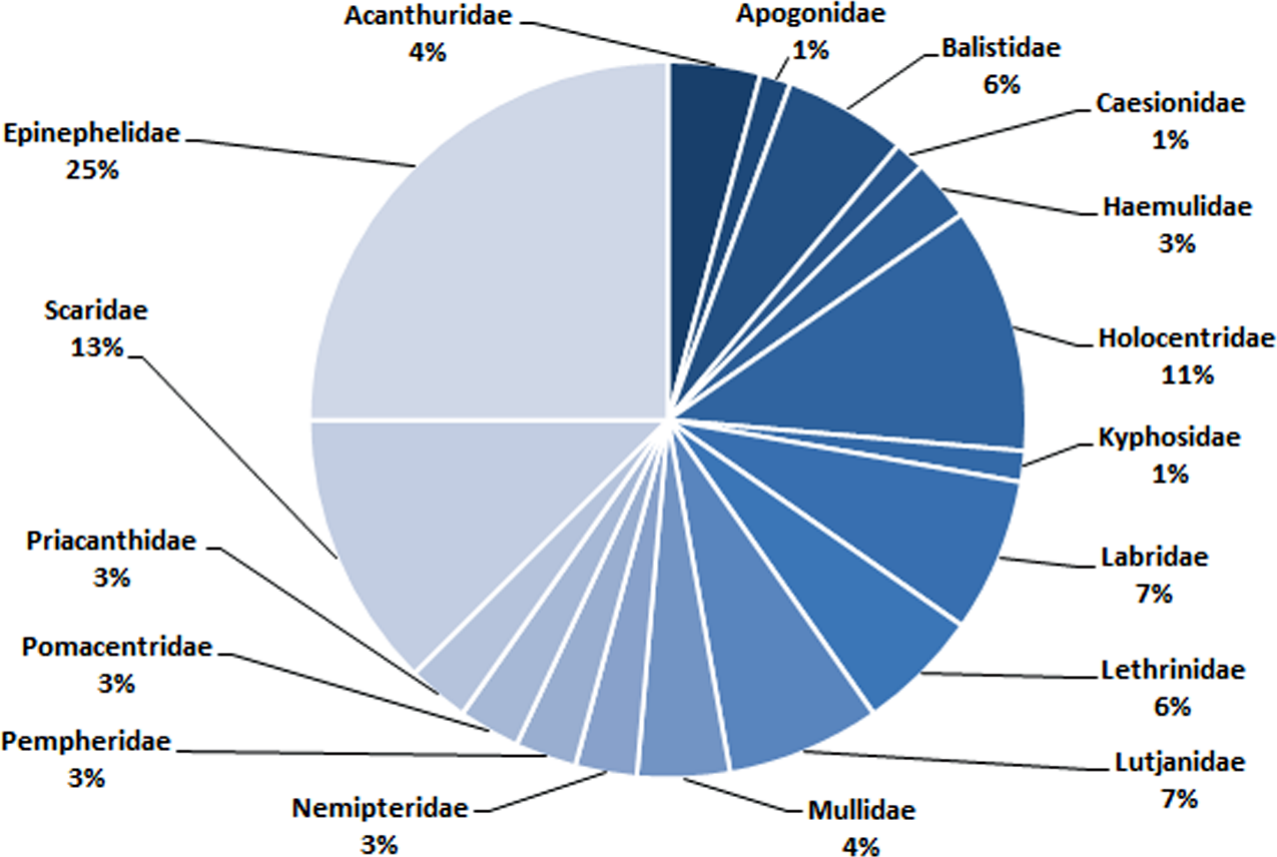
The composition (family) of commercially important reef fishes found in surrounding waters of Weh Island.

In total, 72 species belonging to 32 genera in 17 families were recorded in this study (Table 1). Epinephelidae was the most abundant family found in the Weh Island landing sites containing 18 species belonging to 3 genera (25%), followed by Scaridae (9 species from 2 genera (13%)), Holocentridae (8 species from 3 genera (11%)), Labridae (5 species from 5 genera (7%)) and Lutjanidae (5 species from 1 genus (7%). Family Apogonidae (1 species from a single genus (1%)) and Kyphosidae (1 species from a single genus (1%)) were the least abundant (Table 1, Fig. 6).

### Conservation and trade status

Based on the IUCN categories, of the 72 species sampled in Weh Island, 61 species are categorised as Least Concern (85%), one species (1%) is Data Deficient, ten species (14%) categorized as Not Evaluated (NE) while none (0%) are deemed to be under Near Threatened category. However, two species showed decreasing population trend (*L. decussatus* and *V. albimarginata*) (http://www.iucnredlist.org, accessed 13 April 2020). None of the 72 identified species has been evaluated by CITES (http://www.cites.org/, accessed 13 April 2020).

## Discussion

### Establishment of DNA barcode for Weh Island Reef Fishes

This study has demonstrated the utility of DNA Barcoding to complement the morphological identification of 72 reef fish species from Weh Island. The establishment of a reliable DNA barcode reference records for commercial reef fishes in Weh island has been initiated, contributing to the DNA barcode library of Indonesian marine fishes and the worldwide DNA barcode entries in general. This baseline database is critical for the future fisheries management on this island.

Database sequence similarity and genetic distance comparisons with voucher reference supported the accurate identification of the 72 putative species. The exact or near matches (98% to 100%) identity with reference DNA libraries both in BLAST and the BOLD Identification System (IDS) is regarded as firm evidence in the success of the DNA barcoding approach (Alcantara & Yambot, 2016; Bhattacharjee et al., 2012; Cerutti-Pereyra et al., 2012; Filonzi et al., 2010; Joly et al., 2014; Kress & Erickson, 2012; Ratnasingham & Hebert, 2007; Ward, 2009; Zhang & Hanner, 2012). Species delineation was also supported by ABGD and the monophyletic clustering on the NJ tree of all morphologically recognized species.

The average genetic distances between individuals within species, genera, and families were 0.24%, 11.16%, and 19.01%, respectively, in general agreement with previous reports of marine fishes. For example, the average genetic distances of Australian fishes within species, genera, and families were 0.39%, 9.93%, and 15.46%, respectively (Ward et al., 2005); intra-species distance of South Africa and Australia fishes were 0.21% and 0.28%, respectively (Zemlak et al., 2009); Indian marine fishes were 0.30% (conspecifics), 6.60% (congenerics) and 9.91% (confamilial) (Lakra et al., 2011); divergence of Indo-Pacific coral reef fishes was 1.06% (Hubert et al., 2012); South China Sea fishes divergence were 0.32%, 17.26% and 20.10% within species, genera, and families, respectively (Wang et al., 2012); mean intergeneric distance of fishes from the Caribbean and western central Atlantic regions was 16.6% (Weigt et al., 2012); within-species genetic distances of Indo-West Pacific coral reef fishes was 1.12% (Hubert et al., 2017); genetic distances of fishes from Rongchey Bay, China were 0.21% (conspecifics), 5.28% (congenerics), and 21.30% (confamilial) (Wang et al., 2018); and shore fishes in French Polynesia were 0.66% within species and 12.28% within genera (Delrieu-Trottin et al., 2019). These conspecific genetic distances of less than 2% are in agreement with the species delimitation threshold as proposed by Ward (2009) which further supports the monophyly of each presumed species in this study.

As expected, and in concordance with previous studies a marked elevation of conspecifics versus congeneric genetics distances were observed although the values varied considerably. A 20–30 fold difference was observed in the previous studies listed above which are similar to the divergence values reported by Steinke et al. (2009) (15X), Jaafar et al. (2012) (28X), and Bingpeng et al. (2018) (31X). However, the current study showed a higher value of 46.5-fold. This may be attributable to several factors. Firstly, the conspecifics were also ‘con-population’ ie. from the same population. Although sampled from different landing sites, the populations were most likely a single population due to the proximity of all the landing sites. Previous studies may have represented conspecifics sampling from different geographical populations, with higher potential for genetic variation, consequently increasing the conspecific distance, reflected by the narrower gap between the conspecific versus congeneric distances. However, this factor does not explain the discrepancy as the conspecific distances in our study were of the same magnitude in the current and previous studies. Thus, the elevated comparisons are likely attributable to higher between-species distances i.e., our study sampled a lower number of closely related species.

The “barcoding gap” analysis also supported the delineation of the samples into 72 taxa. Even though there is no universal threshold to delineate species, Meier, Zhang & Ali (2008) defined a barcoding gap as the difference between the smallest congeneric and maximum conspecific divergence. The barcode gap results offer yet another explanation for the elevated congeneric to conspecific differences in our study. As observed (Table 3), when the nearest neighbor is not a closely related species (intra-genera) but belonged to another genus or family, the genetic distance was high (above the mean) and therefore caused biasness in the expected NN values. Furthermore, since the number of species sampled were much lower (72 from the 350 morphologically documented fish species) than the record from previous studies (Campbell et al., 2005; Campbell et al., 2007; Rudi et al., 2008; Rudi et al., 2012b), it is likely that many “true” nearest neighbours were not sampled, thus inflating the congeneric mean.

Reef fishes are among one the most taxonomically challenging groups due to their astonishing diversity of morphological characteristics and colouration, often environmentally influenced (Duarte et al., 2017; Hubert et al., 2010). Their morphometric traits may also change during the stages of ontogenetic development (Bingpeng et al., 2018). Furthermore, traditional morphological classification methods (especially colour) depend on well-preserved samples which are frequently lacking from samples collected from landing sites and fish markets. The majority of fishermen in Weh Island rely on traditional chilling methods for fish preservation (e.g., icing the fish with ice flakes or ice tubes) which often result in loss of colouration and often incomplete/damaged morphological characters, thus making taxonomic identification more complicated. However, there appears to be no issue in accurate morphological identification of the samples. One obvious reason for the perfect match in morphological and molecular analysis is the familiarity with these common commercial species of the island.

### Conservation and trade status

At a glance, the conservation status for each of the 72 barcoded species does not raise any cause for “alarm” with 85%, 14% and 1% categorized into LC, NE and DD, respectively. The majority (LC) do not appear to require any additional protection as required for Critically Endangered, Endangered, Vulnerable or Near Threatened categories (IUCN, 2012). However, sustainable considerations are needed in exploiting the species listed in NE and DD categories as these have no or limited biological, ecological or distributional information for a precise assessment to be made using the IUCN criteria (Sadovy de Mitcheson et al., 2013). Until such data is available and conservation status verified, it would be prudent to accord these group careful attention, at least until their status are evaluated (IUCN, 2012). Disregarding the management of LC category is also “unsafe” as they make up 80% of the bony fish and therefore the fisheries of this area (IUCN, 2019). In coral reef fisheries, “fishing through the food web” is a common fishing practice and Weh Island fisheries is no exception. The fishermen catch both high and low trophic species since all fish species have a market value (Darling & D’Agata, 2017). The dominant families landed are: Epinephelidae, Scaridae, Holocentridae, Labridae, and Lutjanidae. Epinephelidae (groupers) and Lutjanidae (snappers) are at the highest trophic level and the most valuable. In Aceh, the price of one kg grouper may reach 7–10 USD and therefore highly prized by the fishermen. They are being threatened on a global scale due to overfishing (Sadovy de Mitcheson et al., 2013). Being predators, their declination from the ocean may lead to imbalance in the trophic chain. Scaridae (parrotfish) are sold at a more affordable price (one kg may cost 2–3 USD) which makes it more accessible to the general population and thus also at a risk of overharvesting. They are low trophic herbivore species feeding on turf algae that grow on reef substrate and hence function to clear new sites for coral larval settlement (Bonaldo & Bellwood, 2009). A decline in herbivores such as the parrotfish would hinder coral replenishment. Bozec et al. (2016) suggested a 10% harvest limitation and a minimum size of 30 cm of parrotfish catches might sustain the health of reefs in the Carribean. Without a sound management plan and immediate implementation, there is a possibility that reef fish catches in this region will go into further decline due to the overexploitation, causing economic losses and long term ecological damage.

In future, DNA barcoding in the different fields of fisheries in Indonesia could be routinely utilized as fishery managers familiarize with the technique and it becomes affordable. Ardura, Planes & Garcia-Vazquez (2013) suggested that DNA barcoding be included in routine surveys and periodical monitoring of catch diversity. Through precise species identification, DNA barcoding could be applied in ecosystem management and conservation, ichthyoplankton identification, seafood forensics, biosecurity, and invasive species detection, predator–prey relationship, seed identification, fingerling survival and health management (Pavan-Kumar et al., 2018). However, with all its advantages, DNA barcoding also has several limitations, among others: this method requires a reliable DNA library so that newly generated sequences could be compared with the existing databases (Taylor & Harris, 2012). Nuclear mitochondrial pseudogenes (numts) that may be coamplified with mtDNA when using conserved universal PCR primers could lead to erroneous estimates of species diversity (Song et al., 2008). Furthermore, the method is inefficient for hybrid detection (Dudu et al., 2016). Occurrence of hybridization has been documented among a broad range of teleost fishes, both freshwater (Scribner, Page & Bartron, 2000) and marine (Montanari et al., 2012; Qu et al., 2018; Yaakub et al., 2006). The COI gene is of mitochondrial origin, typically derived only from the maternal donor. For this reason, any hybrid fish will be inevitably and erroneously identified as its maternal species (Bhattacharya et al., 2016; Linacre et al., 2011). Thus, if the specimen is a hybrid, the COI barcoding result will enable only the identification of the female parent rather than male (Kochzius et al., 2010). In order to allow the precise identification of the parental pair of a hybrid, additional nuclear markers are suggested to be employed (Bhattacharya et al., 2016; Kochzius et al., 2010; Linacre et al., 2011). Montanari et al. (2012) confirmed the hybrid status of the butterflyfish, genus *Chaetodon* through the utilisation of 20 genus-specific microsatellite loci to complement the mtDNA results. Yaakub et al. (2006) utilised a multi-loci approach based on mitochondrial COI gene, nuclear S7 (intron 1) and nuclear copy of mitochondrial (numt) D-loop region to corroborate the identity of *Thalassoma quinquevittatum* as the maternal and *T. jansenii* as the paternal contributor in a study on hybrid specimens (Labridae: *Thalassoma*) collected from Holmes Reef, an isolated oceanic atoll in the Coral Sea. The nuclear copy of the mitochondrial genome (numt) was detected in all putative parental species and hybrids, further supporting the evidence from the nuclear S7 intro 1. In another study (Qu et al., 2018) the nuclear RYR3 marker was found to be an efficient marker to identify first-generation grouper hybrids to assist barcoding in family Epinephelidae.

Finally, results of the present study have contributed significant records for the molecular taxonomy of commercially important reef fishes from Weh Island. Sequences from the study have been deposited in the BOLD Systems websites, establishing a molecular biodiversity catalogue of reef fishes from Weh Island and the Indonesian Archipelago in general. The data could facilitate improved monitoring, conservation, and management of fisheries in this area. When funds permit, more intensive studies should be conducted with a wider coverage and through direct sampling.

## Conclusions

The establishment of a DNA barcode reference library of important reef fishes from Weh Island in the Indonesian Archipelago has been achieved through this study. In total, 230 specimens from 72 species belonging to 32 genera in 17 families were barcoded. There are no overlaps of pairwise genetic variations between conspecific and interspecific comparisons. Epinephelidae (25%), Scaridae (13%), Holocentridae (11%), Labridae (7%) and Lutjanidae (7%) were the dominant family found in Weh Island landing sites, respectively. The study has a significant contribution to the DNA barcode library of Indonesian marine fishes and the worldwide barcode entries in general. It will also contribute towards achieving more efficient monitoring, conservation, and management of fisheries and has a potential role in the aquaculture industry of this region.

##  Supplemental Information

10.7717/peerj.9641/supp-1 Supplemental Information 1 Raw data from Table 1

## Additional Information and Declarations

### Field Study Permissions

The following information was supplied relating to field study approvals (i.e., approving body and any reference numbers): Field experiments were approved by the Faculty of Marine and Fisheries, Syiah Kuala University, Banda Aceh, Indonesia (16030/A2.1/KP/2016).

### Data Availability

The following information was supplied regarding data availability: The sequences are available in a Supplementary File and at BOLD systems (accessions listed in Table 1).
